# Multidisciplinary team management is associated with improved patient-centered outcomes in multiple pulmonary nodules: a prospective observational cohort study

**DOI:** 10.3389/fonc.2026.1771999

**Published:** 2026-04-10

**Authors:** Tiantian Yuan, Lijuan Yuan, Zhimei Wang, Lijuan Cui, Rongmei Song, Yun Wang

**Affiliations:** Department of Cardiothoracic Surgery, Yancheng School of Clinical Medicine of Nanjing Medical University, Yancheng Third People’s Hospital, Yancheng, Jiangsu, China

**Keywords:** health behavior, multidisciplinary team, multiple pulmonary nodules, negative emotion, observational study, self-management

## Abstract

**Background:**

Multiple pulmonary nodules (MPNs) present diagnostic and management challenges, causing patient anxiety and inconsistent clinical recommendations. While multidisciplinary team (MDT) approaches benefit various chronic conditions, their association with improved outcomes for MPNs remains unexplored.

**Methods:**

This prospective observational cohort study with nonrandomized, pathway-based group allocation enrolled 200 consecutive patients with newly diagnosed MPNs at a tertiary center (January–December 2023). Participants received either MDT collaborative management (n=100) or routine care (n=100) based on their healthcare entry point. The MDT intervention integrated risk stratification, specialist coordination, structured education, and psychological support. Primary outcomes were health knowledge and health behavior scores at 6 months, assessed using a validated 20-item lung nodule health knowledge and behavior questionnaire (each domain scored 0–100). Secondary outcomes included disease benefit perception, health literacy, coping strategies, self-management capabilities, quality of life (WHOQOL-BREF), and patient satisfaction.

**Results:**

Baseline characteristics were comparable between groups. At 6 months, the MDT cohort showed significantly higher health knowledge (85.7 ± 6.8 vs 68.5 ± 7.3, P<0.01) and health behaviors (88.3 ± 7.1 vs 71.2 ± 7.8, P<0.01), with large effect sizes (Cohen’s d>2.2). MDT management was associated with greater improvements across all Benefit Finding Scale domains, enhanced positive coping (30.2 ± 3.6 vs 24.8 ± 3.9, P<0.01), reduced negative coping (7.2 ± 2.9 vs 10.8 ± 3.2, P<0.01), and superior health literacy scores. Quality of life improvements favored MDT across all WHOQOL-BREF domains. Patient satisfaction reached 98% with MDT versus 88% with routine care (P = 0.01).

**Conclusion:**

MDT collaborative management is associated with substantially improved patient-centered outcomes for individuals with MPNs, providing evidence that supports further evaluation and implementation of structured multidisciplinary approaches for this complex population.

## Introduction

Multiple pulmonary nodules (MPNs) represent an increasingly common clinical challenge, defined as two or more discrete lung lesions measuring ≤3 cm in diameter (1). These nodules are classified according to their radiographic density into pure ground-glass nodules (pGGNs), mixed ground-glass nodules (mGGNs), and solid nodules, with distinct implications for malignancy risk (2). Among these subtypes, mGGNs demonstrate the highest malignant potential, followed by pGGNs and solid nodules (3). Clinical decision-making is further complicated by the need to distinguish between the dominant nodule—typically the largest or most morphologically suspicious lesion—and secondary nodules that may represent synchronous primary tumors, intrapulmonary metastases, or benign processes (4).

The heterogeneous nature of MPNs poses substantial diagnostic and therapeutic challenges. While initial evaluation relies primarily on clinical presentation and imaging characteristics, the underlying pathology may range from benign inflammatory processes to multiple primary lung cancers or metastatic disease (5, 6). This diagnostic uncertainty often leads to inconsistent clinical recommendations and conflicting specialist opinions, creating significant anxiety for patients and potentially delaying appropriate intervention (7). Moreover, the management complexity extends beyond initial diagnosis, as patients with MPNs frequently require multiple surgical procedures and prolonged surveillance periods, with consequent impacts on lung function and quality of life (8). These challenges underscore the critical importance of establishing systematic approaches to early diagnosis and coordinated intervention for patients with MPNs (9).

Multidisciplinary team (MDT) collaboration has emerged as an evidence-based approach to managing complex medical conditions, bringing together healthcare professionals from diverse specialties to optimize patient care (10). This model facilitates comprehensive assessment, ensures consistency in clinical decision-making, and improves care coordination (11). The MDT approach has demonstrated particular value in enhancing the efficiency, standardization, and quality of clinical interventions across various chronic diseases (12). Current evidence supports MDT implementation in managing diabetes mellitus (13), Parkinson’s disease (14), and various malignancies (15), with documented improvements in clinical outcomes, patient satisfaction, and healthcare resource utilization.

Despite the theoretical advantages of MDT management for MPNs, empirical evidence regarding its effectiveness in this specific population remains limited. The complexity of MPNs—requiring expertise in radiology, pulmonology, thoracic surgery, and oncology—suggests that coordinated multidisciplinary care could address many current management challenges. However, no studies have systematically evaluated whether MDT collaborative health management is associated with improved patient-centered outcomes including health literacy, self-management capabilities, psychological adaptation, and quality of life in this population.

Conceptually, the MDT model for MPNs care may operate through several interlinked pathways. Coordinated specialist input reduces diagnostic uncertainty and conflicting recommendations, which in turn alleviates patient anxiety. Structured education and continuous support enhance health literacy and self-management capabilities, empowering patients to engage more actively with their care. Psychological support facilitates adaptive coping, enabling patients to derive benefit from their illness experience. These mechanisms collectively contribute to improved quality of life and satisfaction. This conceptual framework guided our selection of patient-centered outcomes and informed interpretation of the observed intervention effects.

Therefore, this prospective observational cohort study was designed to evaluate the association between an MDT collaborative health management model compared to routine care and comprehensive patient outcomes in individuals with newly diagnosed MPNs. We hypothesized that structured MDT management would be associated with enhanced health knowledge, improved positive health behaviors, better disease benefit perception and coping strategies, and higher quality of life compared to conventional management approaches.

## Methods

### Study design and setting

This prospective observational cohort study was conducted to evaluate the association between an MDT collaborative health management model versus routine care and patient-centered outcomes in patients with MPNs. The study was performed at the Department of Cardiothoracic Surgery, Yancheng Third People’s Hospital, a tertiary care center affiliated with Nanjing Medical University, between January 2023 and August 2025. The observational design allowed for comparison of two established clinical management pathways without experimental manipulation. Randomized allocation was not feasible in this context because both care pathways were already operational and embedded within distinct clinical workflows; patients presenting through the specialized lung nodule clinic received MDT care as the standard pathway, whereas those identified through routine health examinations received conventional care. Randomly reassigning patients across these established pathways would have disrupted existing clinical operations and raised ethical concerns regarding withholding an available structured care program. The nonrandomized design therefore reflects real-world care delivery while maintaining ethical standards. The study protocol was approved by the Institutional Review Board of Yancheng Third People’s Hospital (Approval No: LunShen-2025-56), and conducted in accordance with the Declaration of Helsinki principles.

Although this study employed a prospective design, formal clinical trial registration was not pursued because the investigation compared two pre-existing, established care pathways without experimental allocation or novel therapeutic intervention. Under applicable local and institutional regulations, observational studies of established care models are not required to be registered as clinical trials. We acknowledge, however, that prospective registration would have further enhanced transparency and encourage future similar studies to consider registration.

### Participant selection and recruitment

#### Study population

Two hundred consecutive patients with newly diagnosed MPNs presented to our institution were enrolled through systematic screening of the thoracic surgery outpatient clinic and health examination center. Patients were allocated to either the MDT collaborative management cohort (n=100) or the routine management cohort (n=100) based on their initial point of entry into the healthcare system and physician referral patterns, with those referred through the specialized lung nodule clinic receiving MDT management and those identified through routine health examinations receiving standard care.

#### Inclusion criteria

Eligible participants met the following criteria:

- Diagnosis of MPNs confirmed by high-resolution computed tomography (HRCT) scan, defined as two or more nodules ≤30 mm in diameter
- Age 18 years or older
- First-time diagnosis without prior treatment for pulmonarynodules
- Ability to provide independent informed consent and cooperate with follow-up procedures
- Eastern Cooperative Oncology Group (ECOG) performance status of 0–2
- Adequate cognitive function to complete questionnaires

#### Exclusion criteria

Patients were excluded if they had:

- Mental health disorders affecting capacity for informed consent or questionnaire completion
- History of malignant tumors at any anatomical site
- Previous diagnosis of pulmonary tuberculosis or active respiratory infection
- Pregnancy or lactation
- Life expectancy less than 12 months due to comorbidconditions
- Current participation in other clinical studies

### Interventions

#### Routine management group (control cohort)

The control cohort received standard clinical management according to institutional protocols. Comprehensive health records were established documenting baseline demographics, clinical characteristics, imaging findings, and treatment recommendations. Management consisted of standardized health examination procedures with physician notification of lung nodule findings, scheduled follow-up appointments at three-month intervals, and telephone contact every two weeks for symptom assessment. Patients received standard printed educational materials about lung nodules and were instructed to seek immediate medical attention for new respiratory symptoms or concerning changes. Routine care visits typically lasted 15–20 minutes per outpatient encounter. Telephone follow-ups were brief (approximately 5–10 minutes) and focused on symptom inquiry without structured educational or psychological components. Patients in this group did not receive dedicated psychological support services, formal group education sessions, or individualized risk stratification beyond standard clinical assessment. No dedicated care coordinator was assigned to this pathway.

#### MDT collaborative health management model (study cohort)

The study cohort underwent structured management through an established MDT framework incorporating multiple specialties and systematic care coordination.

#### MDT team composition and structure

Team formation occurred through formal institutional designation with defined roles and responsibilities. The multidisciplinary team comprised two certified health managers with specialized training in chronic disease management and general practitioners with pulmonary expertise. Leadership was provided by a senior nursing administrator who coordinated care protocols and quality assurance measures. Core team membership included five specialist nurses representing different clinical departments and physician representatives from cardiothoracic surgery, medical oncology, neurology, and diagnostic radiology. Weekly team meetings were conducted to review patient cases, with emergency consultations available as needed. All team members completed standardized training in lung nodule management guidelines and patient communication strategies before study initiation. A summary of the MDT model components, including team composition, roles, intervention elements, frequency, and patient contact points is provided in **Supplementary Table 1**.

#### Risk stratification and management protocols

Systematic screening employed low-dose spiral computed tomography with artificial intelligence-assisted interpretation to categorize nodule characteristics. Management algorithms were implemented based on nodule dimensions and radiographic features. For nodules measuring ≤5 mm, annual surveillance imaging was scheduled. Nodules between 6–10 mm triggered outpatient subspecialty evaluation within four weeks. Lesions measuring 11–30 mm underwent immediate multidisciplinary consultation with development of individualized management plans.

Risk stratification for nodules exceeding 5 mm incorporated comprehensive assessment of imaging characteristics, including ground-glass opacity percentage, margin definition, and growth patterns. Patients categorized as low-to-moderate risk entered structured surveillance protocols extending at least 24 months. Intermediate and high-risk cases received expedited evaluation through the specialized lung nodule clinic, with follow-up intervals of 3–6 months based on individual risk profiles. Extremely high-risk patients underwent comprehensive multidisciplinary assessment incorporating pulmonology, thoracic surgery, medical oncology, and interventional radiology expertise, with consideration of additional factors including smoking history, family cancer history, and symptom burden. All participants in the MDT cohort received the same core intervention components (risk stratification, specialist coordination, structured education, and psychological support access), although the intensity of specific elements (e.g., frequency of specialist consultations, number of psychological support sessions) varied according to individual risk profiles and clinical needs.

#### Patient education and support systems

Communication infrastructure utilized multiple modalities including telephone consultations, secure messaging platforms, and structured group education sessions. A dedicated study coordinator maintained regular contact with participants, conducting standardized assessments at predetermined intervals. Educational materials were developed specifically for the study population, encompassing a comprehensive manual titled “Precautions for Patients with MPNs” that addressed disease pathophysiology, treatment options, lifestyle modifications, and self-monitoring strategies.

Targeted health education sessions were conducted monthly, addressing disease mechanisms, treatment principles, and evidence-based lifestyle interventions. Participants maintained structured health diaries documenting dietary patterns, exercise regimens, and symptom experiences. Psychological support services were integrated throughout the care continuum, with interventions tailored to individual emotional responses and coping mechanisms. Licensed mental health professionals provided cognitive-behavioral interventions for patients experiencing significant anxiety or adjustment difficulties.

### Outcome assessments

#### Primary outcomes

The primary endpoint was the composite improvement in health knowledge and health behavior scores at 6 months post-enrollment. Assessment utilized a validated lung nodule health knowledge questionnaire developed through expert consensus and pilot testing. The instrument comprised two domains evaluating health knowledge and health behaviors, each containing 20 items scored on a 5-point scale (total score range: 0–100 per domain), with higher scores indicating superior outcomes.

#### Secondary outcomes

Disease benefit perception was measured using the validated Benefit Finding Scale (BFS) (16), encompassing six dimensions: acceptance (3 items), family relationships (2 items), world outlook (4 items), personal growth (7 items), social relationships (3 items), and health behaviors (3 items). The 22-item instrument employed 5-point Likert scaling with higher scores reflecting enhanced benefit finding.

Health literacy assessment utilized the Patient Health Literacy Scale (PHLS) (17), evaluating four competency domains: information access abilities (9 items), communication and interaction capabilities (9 items), health improvement motivation (4 items), and financial support willingness (2 items). The 24-item scale used 5-point response options with scores proportional to literacy levels.

Coping strategies were evaluated through the Simplified Coping Style Questionnaire (SCSQ) (18), comprising positive coping (12 items, score range: 0–36) and negative coping (8 items, score range: 0–24) subscales, with higher scores indicating greater utilization of respective coping styles.

Self-management capabilities were assessed via the Adult Health Self-Management Scale (AHSMSRS) (19), incorporating three dimensions across 38 items (total score range: 38–190), with scores directly proportional to self-management competence.

Quality of life measurement employed the World Health Organization Quality of Life-Brief Version (WHOQOL-BREF), assessing four domains through 26 items scored 1–5 (total range: 26–130), with higher scores indicating superior quality of life.

Patient satisfaction was evaluated using an institutionally developed 100-point scale categorized as: dissatisfied (<60), satisfied (60–89), or very satisfied (>89), with total satisfaction calculated as the combined percentage of satisfied and very satisfied responses.

All patient-reported outcome instruments used in this study (BFS, PHLS, SCSQ, AHSMSRS, WHOQOL-BREF) have been previously validated in Chinese populations with demonstrated acceptable internal consistency (Cronbach’s α > 0.70) and construct validity (20, 21). The health knowledge and behavior questionnaire was developed through Delphi expert consensus and pilot-tested in a sample of 30 MPN patients prior to the study, yielding acceptable reliability (Cronbach’s α = 0.85 for knowledge; 0.82 for behavior). Content validity was established through review by a panel of five pulmonary medicine and health education experts, with a content validity index of 0.92. As this instrument has not been previously published, the complete questionnaire is provided in **Supplementary Appendix S1**.

### Data collection procedures

Baseline assessments were conducted within 72 hours of enrollment by trained research personnel blinded to group allocation. Follow-up assessments occurred at 3 and 6 months post-enrollment, with questionnaires administered in quiet, private settings to ensure data quality. Missing data were minimized through immediate review and clarification of incomplete responses. Data integrity was maintained through double data entry and range checks.

### Statistical analysis

Statistical analyses were performed using SPSS software version 20.0 (IBM Corporation, Armonk, NY). Continuous variables conforming to normal distribution were expressed as mean ± standard deviation and compared using independent samples t-tests. Categorical variables were presented as frequencies with percentages and analyzed using chi-square tests. Normality assumptions were verified using Shapiro-Wilk tests. Homogeneity of variance was assessed using Levene’s test prior to all between-group t-test comparisons. Within-group changes from baseline were assessed using paired t-tests. Repeated-measures ANOVA was employed specifically for coping strategy outcomes (SCSQ), where the time × group interaction was of primary interest to assess whether the trajectory of change differed between cohorts over the follow-up period. For other outcomes where the primary comparison was the 6-month between-group difference, independent t-tests were used because these provided a more direct and interpretable test of the study hypothesis. Sphericity was assessed using Mauchly’s test for the repeated-measures analyses, with Greenhouse-Geisser correction applied when the assumption was violated. Effect sizes were calculated using Cohen’s d for continuous outcomes with 95% confidence intervals.

All analyses followed intention-to-treat principles, with last observation carried forward for missing data. Statistical significance was defined as P<0.05 for all comparisons. No adjustments were made for multiple comparisons given the exploratory nature of secondary outcomes. The primary outcome (health knowledge and behavior) was pre-specified and tested at a conventional alpha level; secondary outcomes were designated *a priori* as exploratory and are reported to characterize the breadth of potential associations rather than for confirmatory inference. Applying corrections for multiple comparisons to exploratory endpoints would increase the risk of type II error and potentially obscure clinically relevant signals warranting investigation in future confirmatory studies. Readers should therefore interpret secondary outcome p-values as descriptive rather than confirmatory.

Missing data details: At the 3-month assessment, 3 participants in the routine care group (reasons: 1 relocation, 2 scheduling conflicts) and 2 in the MDT group (reasons: 1 hospitalization for unrelated illness, 1 lost to contact) had incomplete data. At the 6-month assessment, 5 participants in the routine care group (reasons: 2 relocation, 1 withdrawal of consent, 2 scheduling conflicts) and 3 in the MDT group (reasons: 1 hospitalization, 1 withdrawal of consent, 1 scheduling conflict) had incomplete data. LOCF was applied for these participants. No differential patterns in missing data were observed between groups (Fisher’s exact test P = 0.72 at 6 months). A sensitivity analysis excluding participants with imputed data yielded results consistent with the primary analysis.

### Sample size determination

Sample size was calculated based on detecting a clinically meaningful difference of 15 points in health knowledge scores (primary outcome) between groups, with an estimated standard deviation of 25, power of 80%, and two-sided alpha of 0.05. This yielded a requirement of 88 participants per group; enrollment of 100 per group provided adequate power for secondary analyses and anticipated 10% attrition.

## Results

### Participant characteristics and study flow

The study cohort comprised 200 patients with newly diagnosed MPNs who met eligibility criteria and provided informed consent. Of 237 patients initially screened, 37 were excluded (15 had prior malignancy, 12 declined participation, 7 had active tuberculosis, and 3 had inadequate cognitive function) (**Supplementary Figure 1**). Baseline demographic and clinical characteristics demonstrated excellent balance between the two management cohorts (**Table 1**). The mean age was 63.25 ± 4.19 years in the routine care group and 63.32 ± 4.26 years in the MDT group (P = 0.91), with comparable gender distribution (52% male in routine care versus 50% in MDT group, P = 0.78). Nodule morphology showed similar distributions, with pure ground-glass nodules comprising 45% and 44% of cases, mixed ground-glass nodules representing 30% and 32%, and solid nodules accounting for 25% and 24% in the routine and MDT groups, respectively (P = 0.95). No significant differences were observed in smoking history (42% vs 40%, P = 0.77), family cancer history (18% vs 20%, P = 0.72), or baseline pulmonary function parameters between groups.

**Table 1:** Baseline demographic and clinical characteristics of patients with multiple pulmonary nodules according to management strategy.

Characteristic	Routine management (n=100)	MDT collaborative management (n=100)	Statistical test	P-value
Age (years), mean ± SD	63.25 ± 4.19	63.32 ± 4.26	t = 0.12	0.91
Sex, n (%)			χ² = 0.08	0.78
Male	52 (52.0)	50 (50.0)		
Female	48 (48.0)	50 (50.0)		
Nodule morphology, n (%)			χ² = 0.10	0.95
Pure ground-glass (pGGN)	45 (45.0)	44 (44.0)		
Mixed ground-glass (mGGN)	30 (30.0)	32 (32.0)		
Solid	25 (25.0)	24 (24.0)		
Smoking history, n (%	42 (42.0)	40 (40.0)	χ² = 0.08	0.77
Family cancer history, n (%)	18 (18.0)	20 (20.0)	χ² = 0.13	0.72

MDT, multidisciplinary team; SD, standard deviation; pGGN, pure ground-glass nodule; mGGN, mixed ground-glass nodule. Note: P-values derived from independent samples t-test for continuous variables and chi-square test for categorical variables.

### Health knowledge and behavior outcomes

**Figure 1:**
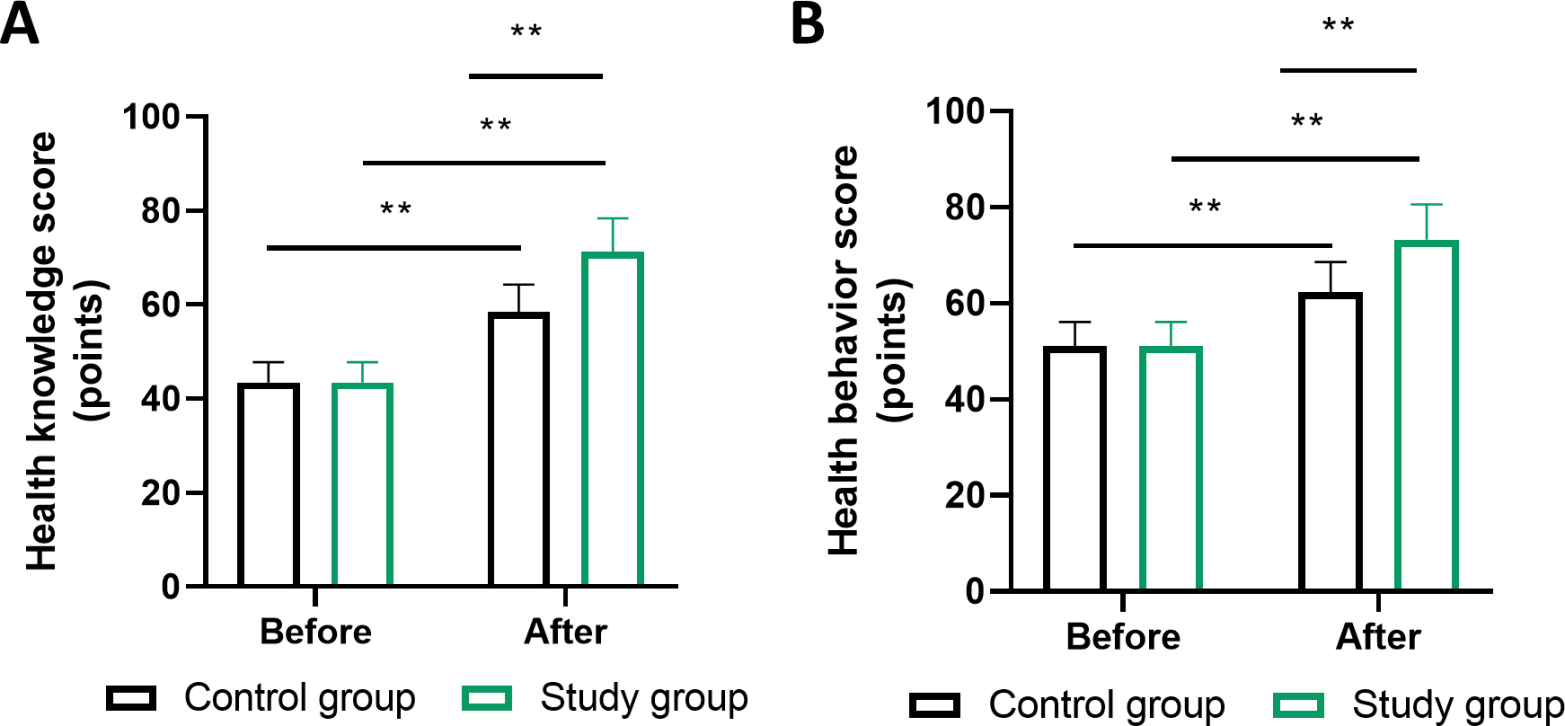
Comparison of health knowledge and health behavior scores between management strategies. **(A)** health knowledge scores and **(B)** health behavior scores at baseline and 6 months post-intervention. Both domains assessed using validated 20-item questionnaires scored on 5-point Likert scales (range 0-100). Statistical comparisons performed using paired t-tests for within-group changes and independent t-tests for between-group differences. ** indicates P<0.01 for between-group comparison at 6 months. n=100 per group. Error bars represent SEM. MDT, multidisciplinary team.

Analysis of health literacy metrics revealed improvements in both cohorts, with significantly greater gains observed in the MDT management group. Baseline health knowledge scores were comparable between groups (routine: 42.3 ± 8.7 vs MDT: 43.1 ± 9.2, P = 0.52). Following the intervention period, both cohorts demonstrated significant improvements from baseline (P<0.01), with the MDT group achieving significantly higher outcomes (routine: 68.5 ± 7.3 vs MDT: 85.7 ± 6.8, P<0.01; **Figure 1**).

Health behavior scores followed a similar trajectory, with no significant baseline differences (routine: 45.6 ± 9.1 vs MDT: 46.2 ± 8.9, P = 0.63) but substantial divergence post-intervention. The MDT cohort demonstrated significantly greater behavioral improvements compared to routine care (routine: 71.2 ± 7.8 vs MDT: 88.3 ± 7.1, P<0.01; **Figure 1**), representing a mean difference of 17.1 points favoring the MDT approach. The effect size for health knowledge improvement was large (Cohen’s d=2.41, 95% CI: 1.98–2.84), as was the effect for health behavior change (Cohen’s d=2.28, 95% CI: 1.86–2.70).

Baseline values, post-intervention values, and within-group changes for all primary and key secondary outcomes are presented in **Supplementary Table 2**.

### Disease benefit and coping mechanisms

**Figure 2:**
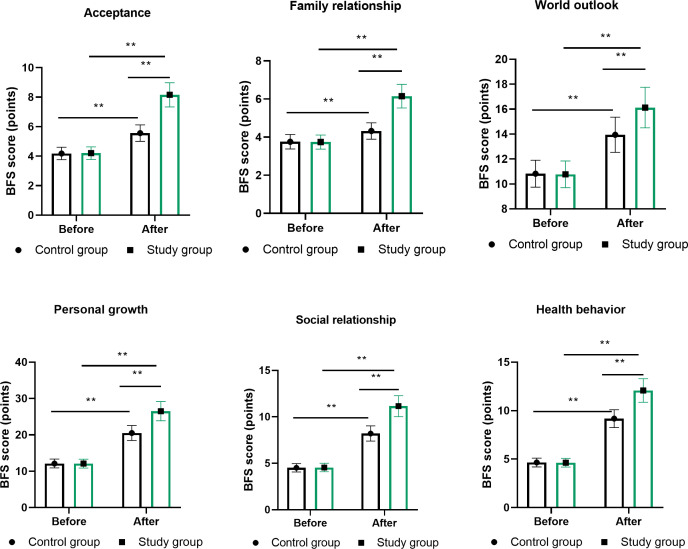
Disease benefit perception across six domains following different management approaches. Benefit Finding Scale (BFS) domains: acceptance (3 items), family relationships (2 items), world outlook (4 items), personal growth (7 items), social relationships (3 items), and health behaviors (3 items). Each item scored 1–5 points on Likert scale. Data shown for baseline and 6-month assessments. ** denotes P<0.01 for between-group differences at 6 months using independent t-tests. n=100 per group. MDT, multidisciplinary team.

The Benefit Finding Scale assessment revealed notable differences in psychological adaptation between management strategies. While baseline BFS scores showed no significant differences across any dimension (all P>0.05), post-intervention assessments demonstrated consistent superiority of the MDT approach across all six domains (**Figure 2**). Acceptance scores increased from 8.2 ± 2.1 to 13.8 ± 1.9 in the MDT group versus 8.3 ± 2.0 to 10.5 ± 2.2 in routine care (P<0.01). Family relationship scores improved from 5.6 ± 1.4 to 8.9 ± 1.2 in the MDT cohort compared to 5.5 ± 1.5 to 7.1 ± 1.3 in controls (P<0.01). Personal growth, representing the largest subscale, showed particularly marked differences, with MDT participants achieving scores of 28.3 ± 3.2 versus 20.1 ± 3.8 in routine care (P<0.01).

**Figure 3:**
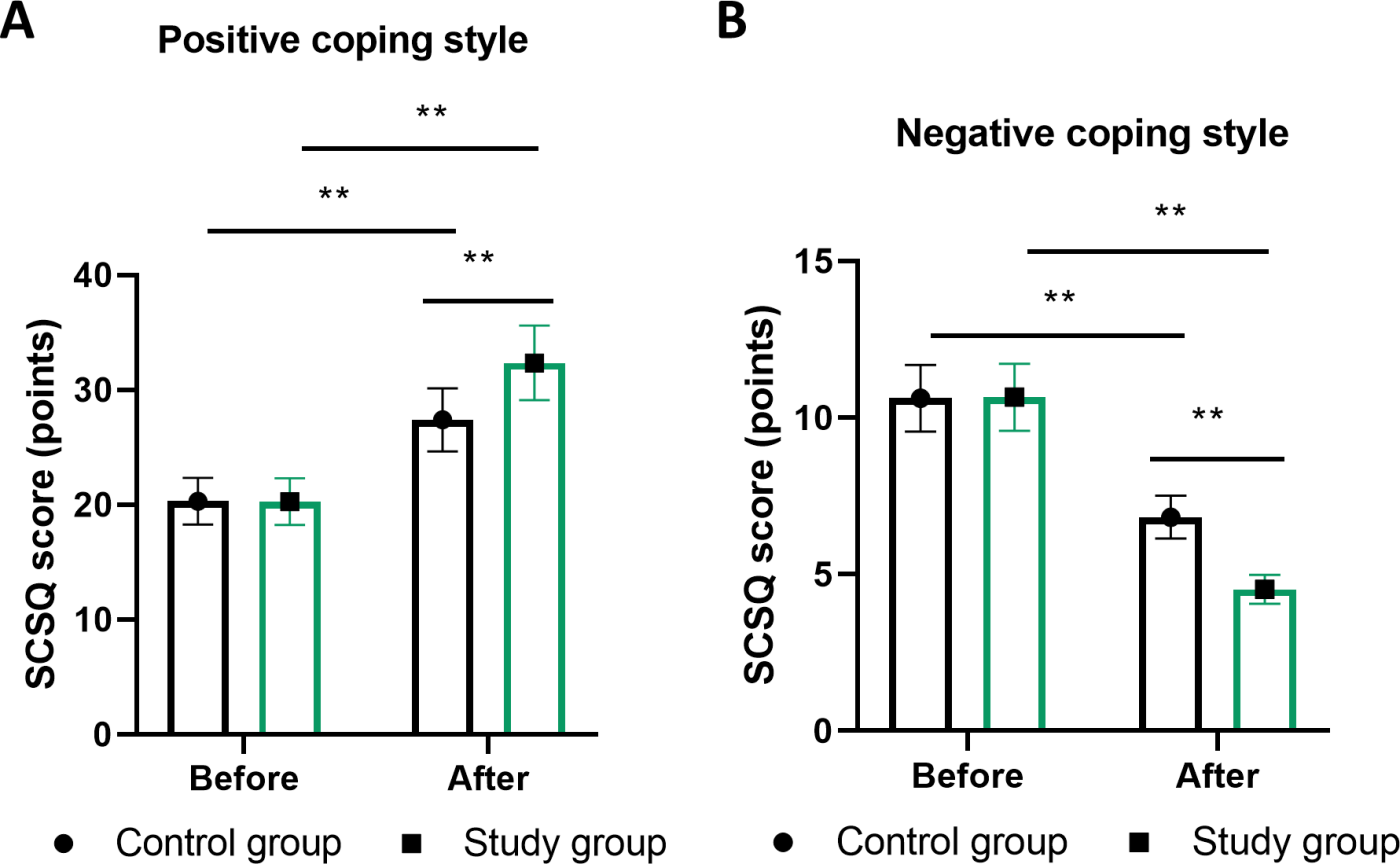
Evolution of coping strategies measured by the Simplified Coping Style Questionnaire. **(A)** positive coping scores (12 items, range 0-36) and **(B)** negative coping scores (8 items, range 0-24) from baseline to 6 months. Higher scores indicate greater utilization of respective coping style. Data presented as mean ± SEM. Statistical analysis using repeated measures ANOVA with *post-hoc* tests. ** denotes P<0.01 for time × group interaction effect. n=100 per group. MDT, multidisciplinary team.

Coping style assessments through the SCSQ revealed meaningful shifts in psychological adaptation strategies. Positive coping behaviors increased significantly in both groups, though the magnitude of change was substantially greater with MDT management (baseline: 18.5 ± 4.2 to post-intervention: 30.2 ± 3.6) compared to routine care (baseline: 18.7 ± 4.1 to post-intervention: 24.8 ± 3.9, P<0.01; **Figure 3**). Conversely, negative coping patterns decreased more substantially in the MDT group (baseline: 15.3 ± 3.8 to post-intervention: 7.2 ± 2.9) versus routine management (baseline: 15.1 ± 3.9 to post-intervention: 10.8 ± 3.2, P<0.01).

### Health literacy and self-management capabilities

**Figure 4:**
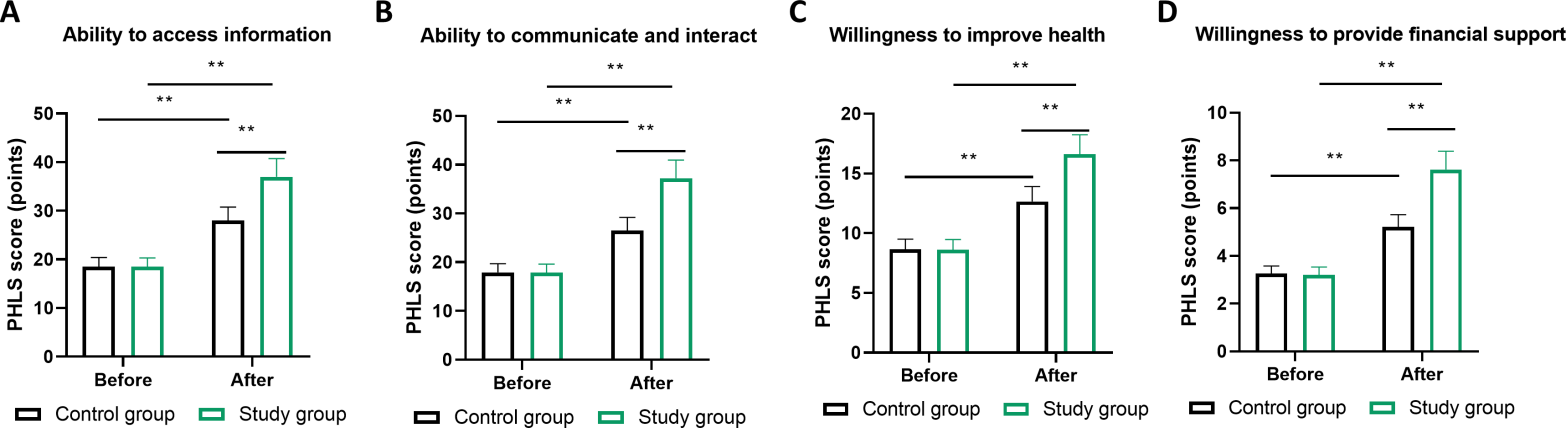
Health literacy competencies evaluated through four domains of the Patient Health Literacy Scale. **(A)** ability to access information (9 items), **(B)** ability to communicate and interact (9 items), **(C)** willingness to improve health (4 items), and **(D)** willingness to provide financial support (2 items). All items assessed using 5-point Likert scales. Measurements obtained at baseline and 6 months post-intervention. ** indicates P<0.01 for difference between MDT collaborative management and routine management at 6 months (independent t-tests). n=100 per group. Error bars represent SEM.

Patient health literacy, assessed through the PHLS, demonstrated improvements across all evaluated domains. The ability to access health information showed the most notable enhancement in the MDT group, with scores increasing from 22.3 ± 5.1 to 38.7 ± 4.3, compared to 22.5 ± 5.0 to 30.2 ± 4.8 in routine care (P<0.01; **Figure 4**). Communication and interaction capabilities similarly improved, with MDT participants achieving post-intervention scores of 39.2 ± 4.1 versus 31.5 ± 4.6 in controls (P<0.01). Willingness to improve health and provide financial support for care also showed significant between-group differences favoring the MDT approach (P<0.01 for both domains).

**Figure 5:**
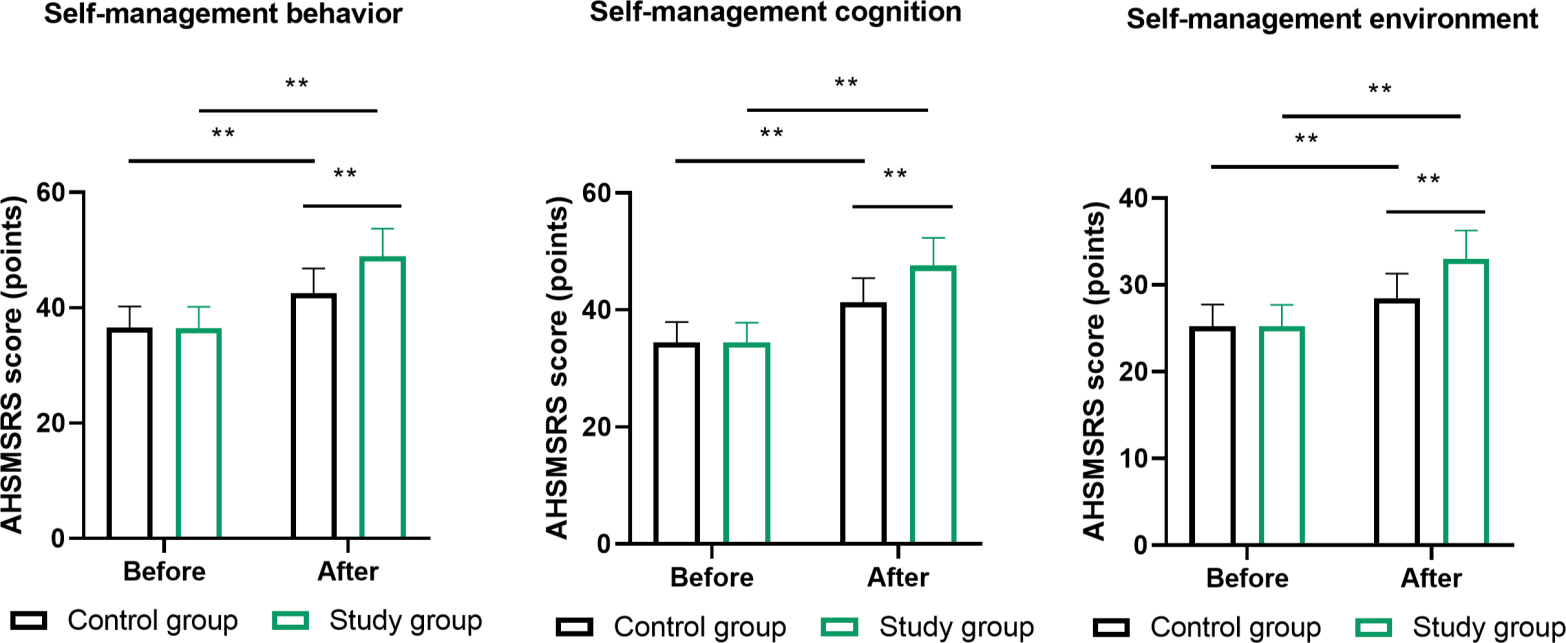
Self-management competencies across three dimensions of the Adult Health Self-Management Scale. Self-management behaviors, self-management cognition, and self-management environment dimensions. Total scale comprises 38 items with composite score range 38-190. Assessments conducted at baseline and 6 months. ** indicates P<0.01 for between-group differences at 6 months using independent t-tests. n=100 per group. Error bars represent SEM. MDT, multidisciplinary team.

Self-management competencies, measured through the AHSMSRS, revealed notable enhancements across all three evaluated dimensions. The MDT cohort demonstrated superior improvements in self-management behaviors (post-intervention: 68.9 ± 7.2 vs 52.3 ± 8.1, P<0.01), self-management cognition (post-intervention: 71.2 ± 6.8 vs 54.7 ± 7.9, P<0.01), and self-management environment (post-intervention: 45.3 ± 5.1 vs 35.8 ± 5.7, P<0.01; **Figure 5**). The composite self-management score improved by 67.8% in the MDT group versus 38.2% in routine care.

### Quality of life outcomes

**Figure 6:**
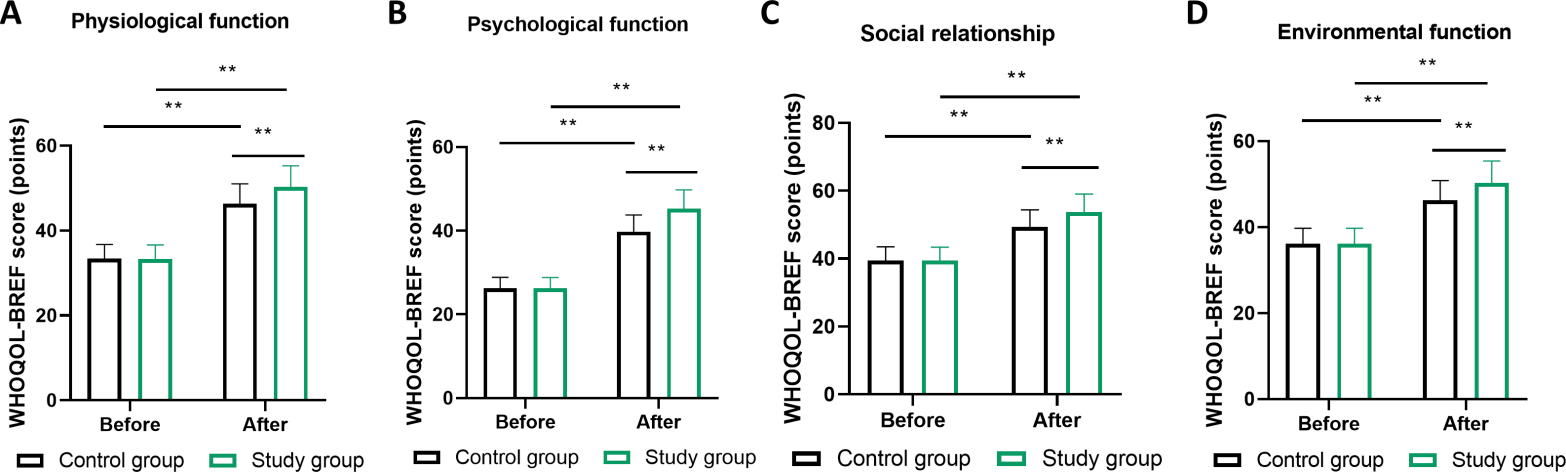
Quality of life outcomes across four domains of the WHOQOL-BREF instrument. **(A)** physiological function, **(B)** psychological function, **(C)** social relationships, and **(D)** environmental function domains. Scale contains 26 items scored 1-5 (total range 26-130), with higher scores indicating superior quality of life. Data collected at baseline and 6 months post-intervention. ** represents P<0.01 for difference between management strategies at 6 months (independent t-tests). n=100 per group. Error bars denote SEM. MDT, multidisciplinary team; WHOQOL-BREF, World Health Organization Quality of Life-Brief Version.

Quality of life assessments using WHOQOL-BREF demonstrated broad benefits associated with MDT management across all evaluated domains (**Figure 6**). Physiological function scores improved from 18.2 ± 3.9 to 25.8 ± 3.2 in the MDT group compared to 18.4 ± 3.8 to 22.1 ± 3.5 in routine care (P<0.01). Psychological function showed particularly notable improvements, with MDT participants achieving scores of 26.3 ± 3.1 versus 21.7 ± 3.4 in controls (P<0.01). Social relationship quality improved from 10.8 ± 2.3 to 14.9 ± 2.0 in the MDT cohort versus 10.9 ± 2.2 to 12.5 ± 2.1 in routine management (P<0.01). Environmental function scores similarly favored the MDT approach (post-intervention: 28.7 ± 3.3 vs 24.2 ± 3.6, P<0.01).

### Patient satisfaction and acceptability

Patient satisfaction assessments revealed marked differences in perceived care quality between management approaches (**Table 2**). The MDT cohort achieved a total satisfaction rate of 98.0%, with 40.0% reporting very satisfied and 58.0% satisfied ratings. In contrast, routine care yielded an 88.0% total satisfaction rate, with 32.0% very satisfied and 56.0% satisfied (χ²=7.68, P = 0.01). Dissatisfaction rates were notably lower in the MDT group (2.0%) compared to routine care (12.0%). Qualitative feedback indicated that MDT participants particularly valued the comprehensive care coordination, enhanced communication, and psychological support components of the intervention.

**Table 2:** Patient satisfaction outcomes at 6 months post-intervention.

Satisfaction category	Routine management n (%)	MDT collaborative management n (%)
Very satisfied (score >89)	32 (32.0)	40 (40.0)
Satisfied (score 60-89)	56 (56.0)	58 (58.0)
Dissatisfied (score <60)	12 (12.0)	2 (2.0)
**Total satisfaction rate**	**88 (88.0)**	**98 (98.0)**

MDT, multidisciplinary team. Note: Total satisfaction rate represents combined percentage of very satisfied and satisfied responses. Between-group comparison: χ² = 7.68, P = 0.01. Satisfaction assessed using a validated 100-point institutional scale administered by independent evaluators.Bold values indicate the total satisfaction rate, which represents the combined percentage of very satisfied and satisfied responses.

No significant adverse events related to either management approach were observed during the study period. Adherence to follow-up assessments exceeded 95% in both groups, with no significant between-group differences in retention rates.

## Discussion

This prospective observational cohort study provides evidence suggesting that MDT collaborative health management is associated with significantly improved multiple patient-centered outcomes in individuals with MPNs compared to routine care. Our findings indicate improvements in health literacy, self-management capabilities, psychological adaptation, and quality of life, with effect sizes exceeding those typically reported in chronic disease management interventions. These results address a critical gap in the literature regarding optimal care delivery models for this increasingly prevalent and clinically complex population.

The observed improvements in health knowledge and behavior scores represent meaningful changes in patient engagement with their condition. The MDT cohort achieved mean improvements of 42.6 points in health knowledge and 42.1 points in health behaviors, compared to 26.2 and 25.6 points respectively in routine care. These differences, with large effect sizes (Cohen’s d > 2.2), exceed improvements reported in previous MDT interventions for other chronic conditions. Muhammad et al. reported more modest gains in knowledge scores among heart failure patients receiving MDT-supervised social support programs (22), while our results show nearly double the magnitude of improvement. This enhanced effectiveness likely reflects the comprehensive nature of our intervention, which integrated structured education, personalized risk stratification, and continuous multidisciplinary support tailored specifically to the unique challenges of MPNs management.

The magnitude of the observed effect sizes warrants comment. Several factors may explain the large differences observed. First, baseline health literacy levels in our population were notably low (mean scores ~42–46 out of 100), reflecting limited prior exposure to structured health education regarding pulmonary nodules, which provided substantial room for improvement. Second, routine care in our setting was relatively fragmented, lacking the coordinated educational and psychological components central to the MDT model, thereby widening the difference between groups. Third, the MDT intervention was intensive, involving monthly group education, dedicated care coordination, and psychological support—a high cumulative dose of interaction that may amplify effects compared to lower-intensity MDT models. While these factors may explain the observed magnitudes, we acknowledge that the effect sizes (Cohen’s d > 2.2, 95% CI: 1.86–2.84) should be interpreted cautiously. These results may not generalize to settings with higher baseline health literacy, more structured usual care, or lower-intensity MDT implementation. Future studies across diverse healthcare contexts will be important to establish the typical range of MDT effects in this population.

Interpreting the clinical significance of these improvements is limited by the absence of established minimal clinically important differences (MCIDs) for the health knowledge and behavior questionnaire in the pulmonary nodule population. While our sample size calculation was based on a 15-point difference as clinically meaningful, no anchor-based or distribution-based MCID has been formally derived for this instrument. The large effect sizes and 95% confidence intervals provide an alternative metric for gauging the magnitude of differences, but we acknowledge that the extent to which these statistical differences translate into clinically meaningful changes in patient behavior and outcomes warrants further investigation. Future studies should prioritize establishing MCIDs for patient-reported outcome measures used in this population to facilitate more precise interpretation.

Notably, the routine care group also demonstrated statistically significant improvements from baseline, with health knowledge increasing by 26.2 points and health behavior by 25.6 points—both exceeding the 15-point threshold used in our sample size calculation. Several factors may account for this improvement. First, repeated exposure to healthcare encounters during the surveillance period likely provided informal health education through clinical interactions. Second, the Hawthorne effect from study participation and periodic questionnaire completion may have heightened health awareness in both groups. Third, regression to the mean from low baseline scores may have contributed to improvements regardless of the intervention received. Fourth, the standard printed educational materials provided to all participants may have had a greater effect than anticipated. These control group improvements do not diminish the incremental benefit of MDT management, which produced substantially greater gains across all outcomes, but they do suggest that structured clinical follow-up itself carries some therapeutic value and that the net benefit attributable specifically to MDT coordination should be interpreted in this context.

The psychological benefits observed in our study merit particular attention. Patients receiving MDT management demonstrated notable improvements across all domains of the Benefit Finding Scale, with the personal growth domain showing the most marked enhancement. This finding aligns with research by Liu et al., who demonstrated that MDT combined with psychological support enabled terminal cancer patients to derive meaning and obtain comprehensive social support from their illness experience (23). However, our results extend these observations by demonstrating that psychological benefits can be achieved even in patients with uncertain diagnoses, where the ambiguity itself represents a significant source of distress. The shift from negative to positive coping strategies observed in the MDT group suggests that coordinated multidisciplinary support helps patients develop adaptive responses to diagnostic uncertainty, transforming a potentially overwhelming experience into an opportunity for personal growth and enhanced health awareness.

Quality of life improvements across all WHOQOL-BREF domains in the MDT group reflect the broad nature of this intervention’s impact. While previous studies have documented quality of life benefits from MDT management in established cancer populations (24), our findings suggest that these benefits extend to patients in the diagnostic and surveillance phase of care. The particularly strong improvements in psychological and social relationship domains suggest that MDT management addresses not only the medical aspects of MPNs but also the broader psychosocial challenges that accompany diagnostic uncertainty. The integrated approach, combining medical expertise with psychological support and peer interaction through group education sessions, appears to create a supportive ecosystem that enhances overall wellbeing beyond what traditional medical management alone can achieve.

Our findings regarding health literacy and self-management capabilities have important implications for long-term outcomes. The superior improvements in information access abilities and communication skills observed in the MDT group suggest that this model better prepares patients for the complex decision-making often required in MPNs management. Millar et al. emphasized that MDT approaches are crucial for promoting health literacy (25), and our results provide quantitative evidence supporting this assertion in the context of pulmonary nodule management. The enhanced self-management capabilities developed through MDT care may be particularly valuable given the prolonged surveillance periods often required for MPNs, where patient adherence and self-monitoring play critical roles in detecting clinically significant changes.

The 98% satisfaction rate achieved in the MDT group, compared to 88% in routine care, reflects patients’ recognition of the value of coordinated care. This finding is consistent with Walter et al., who reported high patient satisfaction with MDT approaches in managing complex medical conditions (26). The low dissatisfaction rate of only 2% in the MDT group suggests that the additional time and resource investment required for multidisciplinary coordination is valued by patients and does not create undue burden or complexity from their perspective.

Several mechanisms likely contribute to the observed differences with MDT management. First, the systematic risk stratification and evidence-based management protocols ensure consistency in clinical decision-making, reducing the confusion and anxiety that often result from conflicting specialist opinions. Second, the integration of multiple disciplines from the outset of care prevents the fragmentation commonly experienced in routine care, where patients may receive sequential rather than coordinated specialist consultations. Third, the structured education and psychological support components address the information and emotional needs that are often overlooked in traditional biomedical care models. Finally, the regular team meetings and systematic follow-up procedures ensure that patient concerns are promptly addressed and management plans are dynamically adjusted based on evolving clinical and psychosocial needs.

It is important to note that this study focused exclusively on patient-reported outcomes and did not evaluate clinical or process-related endpoints such as diagnostic yield, time to definitive diagnosis, malignancy detection rates, or surgical intervention rates. While patient-reported outcomes are critical for understanding the patient experience and have intrinsic value, the absence of clinical endpoints limits the ability to assess whether the observed improvements in health literacy and self-management translate into tangible clinical benefits such as improved adherence to imaging surveillance schedules or earlier detection of malignant transformation. Future studies should incorporate both patient-reported and clinical outcomes to provide a more complete picture of MDT effectiveness.

The reliance on unblinded, subjective patient-reported outcomes introduces the possibility of expectancy effects and social desirability bias. Participants in the MDT group, who received more intensive and visible care, may have reported more favorable outcomes partly because of their awareness of the enhanced intervention. Similarly, social desirability effects may have led participants to report outcomes that align with perceived expectations of their care team. While blinding was not feasible given the fundamentally different care experiences between groups, we note that the consistency of findings across multiple independent validated instruments spanning diverse outcome domains (health knowledge, coping, quality of life, satisfaction) partially mitigates, but does not eliminate, this concern. The use of independently administered questionnaires by trained research personnel also helps to reduce, though not completely avoid, assessment bias.

The implications of our findings extend beyond individual patient care to health system organization and policy. As lung cancer screening programs expand globally, the incidence of MPNs detection will inevitably increase, creating substantial demands on healthcare systems (27, 28). Our results suggest that investing in MDT infrastructure for managing these patients may yield potential dividends through improved patient outcomes, enhanced satisfaction, and potentially more efficient resource utilization through coordinated care. The growing adoption of low-dose CT screening programs, as recommended by major international guidelines, will generate an expanding population of patients with incidentally detected pulmonary nodules who require systematic evaluation and longitudinal monitoring. MDT-based care models may offer a structured framework for managing this anticipated increase in clinical volume while maintaining quality of care and addressing the informational and psychological needs that are often underserved in fragmented care pathways. The observational nature of our study, comparing two existing care pathways, demonstrates that MDT implementation is feasible within current healthcare structures and does not require extensive system redesign.

China’s particular context, with the world’s highest burden of lung cancer and a five-year survival rate of only 16.1% (28), makes effective MPNs management especially critical. The success of the MDT model in our Chinese hospital setting suggests that this approach could be valuable in other healthcare systems facing similar challenges with growing numbers of patients with incidental pulmonary nodules requiring systematic evaluation and management (29). The structured protocols and clear role definitions within our MDT model provide a framework that could be adapted to diverse healthcare settings, from large academic centers to community hospitals.

However, the feasibility of implementing MDT-based models in low-resource settings warrants consideration. The MDT approach described in this study requires dedicated specialist personnel, regular team meetings, and infrastructure for structured education and psychological support—resources that may not be readily available in community-level hospitals or resource-constrained healthcare systems. While MDT care has the potential to reduce disparities by ensuring standardized, high-quality management regardless of individual provider expertise, it could paradoxically create access barriers if available only at well-resourced tertiary centers. Adaptive implementation strategies, such as hub-and-spoke models with remote specialist consultation, task-shifting to trained nurses or community health workers, and digital health-enabled MDT coordination, should be explored to extend the benefits of multidisciplinary care to underserved populations.

This study has several limitations that warrant consideration. First, the observational design, while reflecting real-world care delivery, prevents definitive causal inferences about the relationship between MDT management and improved outcomes. Patients were allocated based on their point of entry into the healthcare system rather than randomization, potentially introducing selection bias despite the comparable baseline characteristics between groups. Although baseline demographics and clinical characteristics were well balanced, unmeasured confounders—such as socioeconomic status, baseline psychological distress, health motivation, or prior healthcare-seeking behavior—may have differed systematically between groups and contributed to the observed differences. We did not collect data on comorbidity burden or socioeconomic proxies, which represents a limitation of our baseline assessment. The comparability of baseline scores on all outcome measures, however, provides some reassurance against major confounding, though it does not eliminate this possibility. Second, the single-center setting and relatively homogeneous patient population may limit generalizability to other healthcare contexts or more diverse populations. Third, the six-month follow-up period, while adequate for assessing immediate impacts on patient-reported outcomes, does not capture long-term clinical outcomes such as cancer detection rates, surgical outcomes, or survival. Fourth, we did not perform formal cost-effectiveness analyses, which would be valuable for informing health policy decisions about MDT implementation. Fifth, as discussed above, the lack of blinding and the reliance on subjective patient-reported outcomes may have inflated the observed between-group differences through expectancy effects and social desirability bias; the large effect sizes should therefore be interpreted with appropriate caution. Finally, the use of LOCF for missing data, while the proportion of missing data was low (5% in routine care and 3% in the MDT group at 6 months), may have introduced bias if participants with missing data differed from completers; sensitivity analysis excluding imputed data yielded consistent results.

Future research should address these limitations through multicenter pragmatic studies with longer follow-up periods to evaluate the association between MDT management and clinical outcomes including timely cancer diagnosis, appropriate surgical intervention, and long-term survival. Cost-effectiveness analyses comparing the costs of MDT implementation with potential savings from improved outcomes and reduced healthcare utilization would inform policy decisions. Hybrid effectiveness-implementation studies examining which specific components of the MDT intervention contribute most to improved outcomes could guide more efficient implementation strategies. Additionally, investigation of optimal team composition, meeting frequency, and communication strategies could help refine the MDT model for different healthcare settings and resource constraints. Studies incorporating objective process outcomes (e.g., adherence to surveillance imaging schedules, time to diagnosis, no-show rates) alongside patient-reported outcomes would strengthen the evidence base and address potential bias from unblinded subjective assessments. Finally, propensity score–matched or randomized designs, where feasible, would provide stronger causal evidence for MDT effectiveness in this population.

In conclusion, this study provides evidence suggesting that MDT collaborative health management is associated with meaningfully improved patient-centered outcomes for individuals with MPNs compared to routine care. The broad benefits observed—spanning health literacy, psychological adaptation, self-management capabilities, and quality of life—support further evaluation and broader implementation of structured multidisciplinary approaches for this complex patient population. As healthcare systems worldwide grapple with increasing numbers of patients with incidental pulmonary nodules, our findings suggest that coordinated MDT care represents a promising approach that warrants rigorous evaluation in diverse settings. The success of this model in addressing both the medical and psychosocial challenges of MPNs management offers a promising framework for improving the care of patients facing diagnostic uncertainty in the era of advanced imaging and precision medicine.

## Data Availability

The raw data supporting the conclusions of this article will be made available by the authors, without undue reservation.
