# Supplementary material for: Multidisciplinary team management is associated with improved patient-centered outcomes in multiple pulmonary nodules: a prospective observational cohort study
Source: Front Oncol. 2026 Apr 10;16:1771999. doi: 10.3389/fonc.2026.1771999 (PMC13105896; doi:10.3389/fonc.2026.1771999)
Supplement: Supplementary file 1 [file DataSheet1.docx]

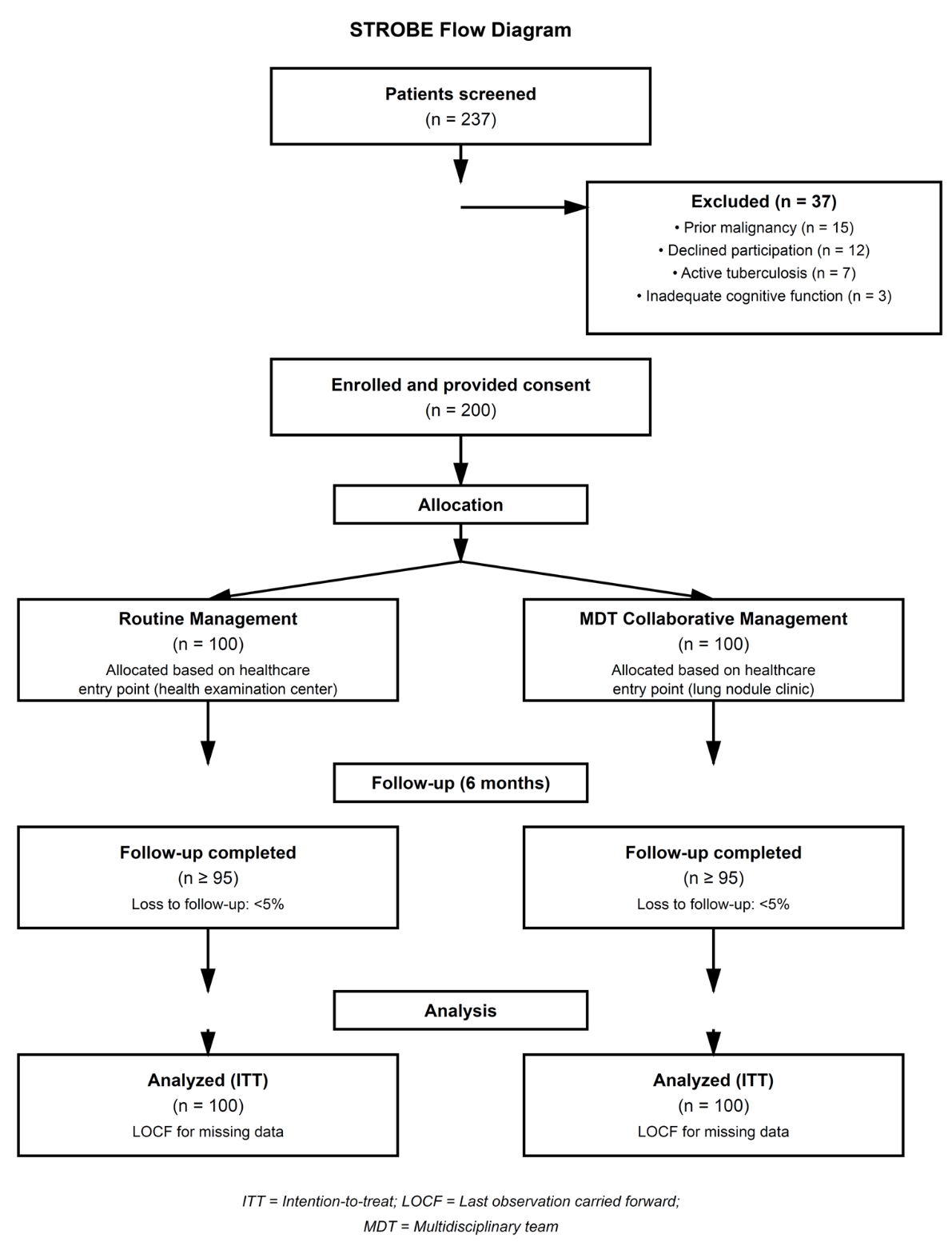


**Supplementary figure 1. STROBE flow diagram for participant enrollment, allocation, follow-up, and analysis.** A total of 237 patients with newly diagnosed multiple pulmonary nodules were screened for eligibility between January and December 2023. After excluding 37 patients, 200 participants were enrolled and allocated to routine management (n=100) or multidisciplinary team (MDT) collaborative management (n=100) based on their healthcare entry point. Follow-up completion exceeded 95% in both groups at 6 months. All enrolled participants were included in the intention-to-treat analysis with last observation carried forward for missing data. ITT, intention-to-treat; LOCF, last observation carried forward; MDT, multidisciplinary team.
