# Supplementary material for: Multidisciplinary team management is associated with improved patient-centered outcomes in multiple pulmonary nodules: a prospective observational cohort study
Source: Front Oncol. 2026 Apr 10;16:1771999. doi: 10.3389/fonc.2026.1771999 (PMC13105896; doi:10.3389/fonc.2026.1771999)
Supplement: Supplementary file 2 [file DataSheet2.docx]

**Supplementary Appendix S1**

**Lung Nodule Health Knowledge and Health Behavior Questionnaire**

**Instrument Overview**

This questionnaire was developed to assess health knowledge and health behaviors among patients with multiple pulmonary nodules (MPNs). The instrument was constructed through a Delphi expert consensus process involving a panel of five pulmonary medicine and health education experts, and subsequently pilot-tested in a sample of 30 MPN patients.

**Psychometric properties:** Content validity index (CVI) = 0.92; internal consistency: Cronbach’s α = 0.85 (health knowledge domain), Cronbach’s α = 0.82 (health behavior domain).

**Structure:** The questionnaire comprises two domains: Health Knowledge (20 items) and Health Behavior (20 items). Each item is rated on a 5-point Likert scale (1 = strongly disagree / never, 5 = strongly agree / always). Domain scores range from 20 to 100, with higher scores indicating greater health knowledge or more positive health behaviors.

**Scoring Instructions**

**Response scale:** 1 = Strongly disagree / Never; 2 = Disagree / Rarely; 3 = Neutral / Sometimes; 4 = Agree / Often; 5 = Strongly agree / Always

**Domain score calculation:** Sum of all 20 item scores within each domain (range: 20–100).

***Note:*** *No items require reverse scoring.*

**Domain 1: Health Knowledge (20 items)**

***Instructions to participants:*** *Please indicate the extent to which you agree with each of the following statements about your understanding of pulmonary nodules. There are no right or wrong answers.*

| **No.** | **Item** | **1** | **2** | **3** | **4** | **5** |
| --- | --- | --- | --- | --- | --- | --- |
| 1 | I understand what pulmonary nodules are and how they are detected. | □ | □ | □ | □ | □ |
| 2 | I can identify the different types of pulmonary nodules (pure ground-glass, mixed ground-glass, and solid nodules). | □ | □ | □ | □ | □ |
| 3 | I understand the relationship between nodule characteristics (size, density, margins) and malignancy risk. | □ | □ | □ | □ | □ |
| 4 | I know the difference between a dominant nodule and secondary nodules in multiple pulmonary nodules. | □ | □ | □ | □ | □ |
| 5 | I understand why regular follow-up imaging (CT scans) is necessary for monitoring pulmonary nodules. | □ | □ | □ | □ | □ |
| 6 | I am aware of the recommended follow-up intervals for pulmonary nodules of different sizes. | □ | □ | □ | □ | □ |
| 7 | I understand the role of low-dose computed tomography in pulmonary nodule surveillance. | □ | □ | □ | □ | □ |
| 8 | I know when surgical intervention may be considered for pulmonary nodules. | □ | □ | □ | □ | □ |
| 9 | I understand the potential causes of pulmonary nodules (benign inflammation, infection, malignancy). | □ | □ | □ | □ | □ |
| 10 | I am aware of the risk factors that increase the likelihood of malignancy in pulmonary nodules (e.g., smoking history, family cancer history). | □ | □ | □ | □ | □ |
| 11 | I understand how artificial intelligence-assisted imaging can help in evaluating pulmonary nodules. | □ | □ | □ | □ | □ |
| 12 | I know the warning signs and symptoms that require immediate medical attention. | □ | □ | □ | □ | □ |
| 13 | I understand the importance of maintaining a health diary to track symptoms and changes. | □ | □ | □ | □ | □ |
| 14 | I am aware of the psychological impact that diagnostic uncertainty from pulmonary nodules can cause. | □ | □ | □ | □ | □ |
| 15 | I understand how lifestyle factors (smoking, diet, exercise) may influence pulmonary nodule outcomes. | □ | □ | □ | □ | □ |
| 16 | I know the benefits and potential risks of diagnostic procedures (e.g., biopsy, bronchoscopy). | □ | □ | □ | □ | □ |
| 17 | I understand the role of the multidisciplinary team in managing multiple pulmonary nodules. | □ | □ | □ | □ | □ |
| 18 | I am aware of the importance of adherence to the recommended surveillance imaging schedule. | □ | □ | □ | □ | □ |
| 19 | I understand the concept of risk stratification in guiding management decisions for pulmonary nodules. | □ | □ | □ | □ | □ |
| 20 | I know where to find reliable health information about pulmonary nodules and lung health. | □ | □ | □ | □ | □ |

**Domain 2: Health Behavior (20 items)**

***Instructions to participants:*** *Please indicate how often you perform each of the following health behaviors related to managing your pulmonary nodule condition. There are no right or wrong answers.*

| **No.** | **Item** | **1** | **2** | **3** | **4** | **5** |
| --- | --- | --- | --- | --- | --- | --- |
| 1 | I attend all scheduled follow-up appointments on time. | □ | □ | □ | □ | □ |
| 2 | I complete all recommended imaging examinations (CT scans) according to the surveillance schedule. | □ | □ | □ | □ | □ |
| 3 | I maintain a health diary recording my symptoms, dietary patterns, and exercise activities. | □ | □ | □ | □ | □ |
| 4 | I actively seek reliable health information about my pulmonary nodule condition. | □ | □ | □ | □ | □ |
| 5 | I communicate openly with my healthcare providers about my symptoms and concerns. | □ | □ | □ | □ | □ |
| 6 | I practice regular physical exercise as recommended by my healthcare team. | □ | □ | □ | □ | □ |
| 7 | I follow dietary recommendations to support my overall lung health. | □ | □ | □ | □ | □ |
| 8 | I have quit smoking or avoid exposure to secondhand smoke. | □ | □ | □ | □ | □ |
| 9 | I avoid known environmental risk factors (e.g., occupational dust, air pollution) when possible. | □ | □ | □ | □ | □ |
| 10 | I take prescribed medications regularly and as directed. | □ | □ | □ | □ | □ |
| 11 | I monitor my respiratory symptoms (cough, shortness of breath, chest pain) and report changes promptly. | □ | □ | □ | □ | □ |
| 12 | I participate actively in health education sessions when available. | □ | □ | □ | □ | □ |
| 13 | I share health information about pulmonary nodules with my family members. | □ | □ | □ | □ | □ |
| 14 | I seek psychological support or counseling when feeling anxious about my condition. | □ | □ | □ | □ | □ |
| 15 | I use stress management techniques (e.g., relaxation, deep breathing) to cope with diagnostic uncertainty. | □ | □ | □ | □ | □ |
| 16 | I maintain a regular sleep schedule to support my overall health. | □ | □ | □ | □ | □ |
| 17 | I limit alcohol consumption as recommended for general health. | □ | □ | □ | □ | □ |
| 18 | I engage in self-monitoring of my weight and general physical condition. | □ | □ | □ | □ | □ |
| 19 | I actively participate in making decisions about my healthcare plan. | □ | □ | □ | □ | □ |
| 20 | I follow up with my healthcare team if I experience any new or worsening symptoms between scheduled visits. | □ | □ | □ | □ | □ |
