# Supplementary material for: Multidisciplinary team management is associated with improved patient-centered outcomes in multiple pulmonary nodules: a prospective observational cohort study
Source: Front Oncol. 2026 Apr 10;16:1771999. doi: 10.3389/fonc.2026.1771999 (PMC13105896; doi:10.3389/fonc.2026.1771999)
Supplement: Supplementary file 3 [file Table1.docx]

**Supplementary Table S1. Summary of the MDT Collaborative Health Management Model Components.**

| **Component** | **Details** | **Frequency / Timing** | **Patient Contact** |
| --- | --- | --- | --- |
| Team Composition | 2 certified health managers, 5 specialist nurses, physicians from cardiothoracic surgery, medical oncology, neurology, diagnostic radiology, senior nursing administrator (team lead) | Ongoing throughout study period | Direct care delivery and coordination |
| MDT Case Review Meetings | Review of patient cases, management plan adjustment, quality assurance | Weekly; emergency consultations as needed | Indirect (informs care plan communicated to patients) |
| Risk Stratification | Low-dose spiral CT with AI-assisted interpretation; categorization by nodule size and imaging characteristics | At enrollment; updated at follow-up imaging | Results communicated to patient with individualized plan |
| Structured Health Education | Group sessions covering disease mechanisms, treatment principles, evidence-based lifestyle interventions; “Precautions for Patients with MPNs” manual provided | Monthly group sessions | Direct: group education sessions |
| Dedicated Care Coordination | Assigned study coordinator for regular contact, standardized assessments, scheduling | Continuous; structured assessments at predetermined intervals | Direct: telephone, secure messaging, in-person |
| Psychological Support | Licensed mental health professionals providing cognitive-behavioral interventions; tailored to individual emotional responses and coping mechanisms | As needed, integrated throughout care continuum | Direct: individual counseling sessions |
| Health Diary Monitoring | Structured diaries for dietary patterns, exercise regimens, symptom experiences | Continuous (patient-maintained) | Reviewed at follow-up visits |
| Follow-up Imaging | Based on risk stratification: ≤5mm annual; 6–10mm outpatient within 4 weeks; 11–30mm immediate MDT consultation | Per risk protocol (3–12 months) | Direct: imaging appointment with results review |
| Average MDT Interaction Intensity | Approximate total: 6 group education sessions, 12+ coordinator contacts, 2–4 specialist visits, psychological support as needed over 6 months | Approximately 20–25 structured contact points over 6 months | Mixed: individual and group formats |

Abbreviations: MDT, multidisciplinary team; CT, computed tomography; AI, artificial intelligence; MPNs, multiple pulmonary nodules. Note: All participants received the same core intervention components, though intensity of specific elements varied based on individual risk profiles and clinical needs.

**Supplementary Table S2. Baseline Values, Post-Intervention Values, and Within-Group Changes for Primary and Key Secondary Outcomes.**

| **Outcome** | **Group** | **Baseline (Mean±SD)** | **6-Month (Mean±SD)** | **Change (Mean±SD)** | **Cohen’s d (95% CI)** |
| --- | --- | --- | --- | --- | --- |
| Health Knowledge | Routine | 42.3±8.7 | 68.5±7.3 | 26.2±9.8 |  |
| (score 0–100) | MDT | 43.1±9.2 | 85.7±6.8** | 42.6±10.1 | 2.41 (1.98–2.84) |
| Health Behavior | Routine | 45.6±9.1 | 71.2±7.8 | 25.6±10.2 |  |
| (score 0–100) | MDT | 46.2±8.9 | 88.3±7.1** | 42.1±9.7 | 2.28 (1.86–2.70) |
| Positive Coping (SCSQ) | Routine | 18.7±4.1 | 24.8±3.9 | 6.1±4.5 |  |
| (score 0–36) | MDT | 18.5±4.2 | 30.2±3.6** | 11.7±4.3 | 1.44 (1.08–1.79) |
| Negative Coping (SCSQ) | Routine | 15.1±3.9 | 10.8±3.2 | −4.3±4.0 |  |
| (score 0–24) | MDT | 15.3±3.8 | 7.2±2.9** | −8.1±3.7 | 1.18 (0.83–1.52) |
| BFS – Acceptance | Routine | 8.3±2.0 | 10.5±2.2 | 2.2±2.3 |  |
|  | MDT | 8.2±2.1 | 13.8±1.9** | 5.6±2.1 | 1.61 (1.24–1.97) |
| BFS – Personal Growth | Routine | 17.8±3.5 | 20.1±3.8 | 2.3±3.6 |  |
|  | MDT | 17.5±3.6 | 28.3±3.2** | 10.8±3.4 | 2.33 (1.91–2.75) |
| Self-Management Total | Routine | 95.2±15.3 | 142.8±18.1 | 47.6±18.5 |  |
| (AHSMSRS, 38–190) | MDT | 96.1±14.8 | 185.4±16.2** | 89.3±17.1 | 2.48 (2.04–2.92) |
| WHOQOL-BREF Psychological | Routine | 17.9±3.5 | 21.7±3.4 | 3.8±3.7 |  |
|  | MDT | 18.1±3.4 | 26.3±3.1** | 8.2±3.3 | 1.41 (1.06–1.77) |

Abbreviations: MDT, multidisciplinary team; SD, standard deviation; CI, confidence interval; SCSQ, Simplified Coping Style Questionnaire; BFS, Benefit Finding Scale; AHSMSRS, Adult Health Self-Management Scale; WHOQOL-BREF, World Health Organization Quality of Life-Brief Version. **P<0.01 for between-group comparison at 6 months (independent t-test). Cohen’s d and 95% CI reported for between-group effect size at 6 months. n=100 per group.

**Supplementary Table S3. Summary of Missing Data by Group and Assessment Timepoint.**

| **Timepoint** | **Routine Care n (%)** | **MDT n (%)** | **Total n (%)** | **Fisher’s P** | **Reasons** |
| --- | --- | --- | --- | --- | --- |
| Baseline | 0 (0%) | 0 (0%) | 0 (0%) | — | — |
| 3-month assessment | 3 (3.0%) | 2 (2.0%) | 5 (2.5%) | 1.00 | Routine: 1 relocation, 2 scheduling. MDT: 1 hospitalization (unrelated), 1 lost to contact |
| 6-month assessment | 5 (5.0%) | 3 (3.0%) | 8 (4.0%) | 0.72 | Routine: 2 relocation, 1 consent withdrawal, 2 scheduling. MDT: 1 hospitalization, 1 consent withdrawal, 1 scheduling |

Note: Missing data were handled using last observation carried forward (LOCF). Sensitivity analysis excluding participants with imputed data yielded results consistent with primary analysis. No differential attrition patterns were observed between groups.
